# Ecological niche modeling predicting the potential distribution of African horse sickness virus from 2020 to 2060

**DOI:** 10.1038/s41598-022-05826-3

**Published:** 2022-02-02

**Authors:** Ayalew Assefa, Abebe Tibebu, Amare Bihon, Alemu Dagnachew, Yimer Muktar

**Affiliations:** 1 Department of Veterinary Medicine, Woldia University, Woldia, Ethiopia; 2 Sekota Dryland Agricultural Research Center, Sekota, Ethiopia

**Keywords:** Ecological epidemiology, Epidemiology

## Abstract

African horse sickness is a vector-borne, non-contagious and highly infectious disease of equines caused by African horse sickness viruses (AHSv) that mainly affect horses. The occurrence of the disease causes huge economic impacts because of its high fatality rate, trade ban and disease control costs. In the planning of vectors and vector-borne diseases like AHS, the application of Ecological niche models (ENM) used an enormous contribution in precisely delineating the suitable habitats of the vector. We developed an ENM to delineate the global suitability of AHSv based on retrospective outbreak data records from 2005 to 2019. The model was developed in an R software program using the Biomod2 package with an Ensemble modeling technique. Predictive environmental variables like mean diurnal range, mean precipitation of driest month(mm), precipitation seasonality (cv), mean annual maximum temperature (^o^c), mean annual minimum temperature (^o^c), mean precipitation of warmest quarter(mm), mean precipitation of coldest quarter (mm), mean annual precipitation (mm), solar radiation (kj /day), elevation/altitude (m), wind speed (m/s) were used to develop the model. From these variables, solar radiation, mean maximum temperature, average annual precipitation, altitude and precipitation seasonality contributed 36.83%, 17.1%, 14.34%, 7.61%, and 6.4%, respectively. The model depicted the sub-Sahara African continent as the most suitable area for the virus. Mainly Senegal, Burkina Faso, Niger, Nigeria, Ethiopia, Sudan, Somalia, South Africa, Zimbabwe, Madagascar and Malawi are African countries identified as highly suitable countries for the virus. Besides, OIE-listed disease-free countries like India, Australia, Brazil, Paraguay and Bolivia have been found suitable for the virus. This model can be used as an epidemiological tool in planning control and surveillance of diseases nationally or internationally.

## Introduction

African horse sickness (AHS) is a non-contagious, highly infectious vector-borne disease of equines caused by the African horse sickness virus (AHSv). The disease severely affects horses, while mules, donkeys, and zebras are less susceptible^1^. The disease was described in early Arabic documents dating back to 1327, in which horses were suffering from an apparent AHS-like disease in Yemen^2^.

Africa is considered the hotspot for the disease because of increased outbreaks that occur each year. The disease was first reported in the early seventeenth century when a major outbreak was observed in 1719 in South Africa. Since then, the African horse sickness virus has become endemic in Africa, stretching from west to east and extending to South Africa^2^. Even though AHS is endemic through most sub-Saharan Africa, with outbreaks occurring regularly, recently, outbreaks have been reported outside of Africa, including Spain, the Middle East and the Indian subcontinent^3^.

AHS outbreaks in endemic areas have a devastating economic and social impact on the economy due to rapid spread, direct mortalities, restriction of animal movements, surveillance and vaccination costs and immediate notification requirement for the World Animal Health Organization^4^. An outbreak of AHS in a disease-free region would have catastrophic effects on equine welfare and industry, particularly for international events such as the Olympic games and restrictions for international trade of racehorses^5^.

The disease is vector-borne, transmitted by midges belonging to the genus Culicoides. Female Culicoides carry the virus from a diseased animal while feeding on blood and transmitting it to healthy animals. Vector abundance and reservoir existence in a particular area play a key role in transmitting the disease^4^. Even though the genus Culicoides species are the most dominant vector for the diseases, other insects can transmit the virus. For example, mosquitoes of the *Aedes, Culex* and *Anopheles* genera or ticks of the *Hyalomma* or *Rhipicephalus* genera are vectors capable of transmitting the virus^2^.

Ecological niche modeling (ENM) is a commonly used technique in ecology to predict species' probable geographic ranges with environmental restrictions. It is quickly becoming the gold-standard method for disease risk mapping. The majority of infectious diseases in animals do not spread evenly. Diseases in livestock and wildlife, on the other hand, are predictable in terms of region, time, and species. Ecological niche modeling tools have been critical in furthering our understanding of disease patterns and variety. For various infectious diseases, reports describing the spatial location of pathogens, disease vectors, or reservoirs are becoming more abundant, high-quality, and publically available .

Many databases of soil composition and structure, landscape composition and structure, climate and geomorphology, and climate and geomorphology, for example, are freely and openly available for mapping diseases in aquatic and terrestrial ecosystems around the world. These characteristics can be combined with disease data to reconstruct or anticipate the regional spread of environmental (e.g., anthrax), vector-borne (e.g., Bluetongue disease), and directly transmitted (e.g., rabies) diseases. On the other hand, these investigations necessitate a fundamental knowledge of Geographic Information Systems, spatial statistics, and a thorough grasp of the biology of the disease system to be modeled^6^.

Ecological niche models can determine vectors and infectious disease distribution in space^7–9^. These models can be used to delineate and predict suitable territories of vector-borne diseases. The study aimed to delineate the global suitability level and distribution of AHSv outbreaks by identifying environmental derived risk factors with ENMs from 2005–2019. Besides, we predicted the future suitability level for the years 2020 to 2040 and 2040 to 2060.

## Material and methods

### African horse sickness outbreak data source

AHS is a notifiable disease in which authorities of member countries of the OIE report any case immediately. Each outbreak case is georeferenced and available at the Global Animal Disease Information System website (EMPRES-i). The reported outbreak comprises information about the locality, region, number of animals affected and other relevant information. For this model, we utilized data downloaded EMPRES database dating back to 2005 to 2019 with 183 georeferenced outbreak reports from Africa (supplementary file 2, Fig. 1 and supplementary file 3).

### Environmental data sources

Variables known to determine the maintenance and circulation of AHS and Culicoides species were determined based on their biological plausibility. The variables comprise topographic and climate variables downloaded from the worldclim 2.1 database^10^.

Climate and topographic variables, including annual maximum and minimum temperature, annual average precipitation, wind speed, solar radiation, elevation and other bioclimatic variables, have been used in the model. These variables are known to determine the spread of the disease circulating in the equine population^11^. Initially, 19 bioclimatic variables and six weather variables were proposed. However, most of the variables were trimmed and only eleven variables were utilized due to multicollinearity. Besides, projected bioclimatic datasets from 2020 to 2040 and 2040 to 2060 were downloaded from the new WorldClim 2.1 database.

### Spatial data handling and management

The GIS data downloaded from different sources had varied projection, spatial resolution, and cell size. These data sets were projected to the same projection system, resampled to the same cell size (2.5 min) and extent. These GIS operations are processed using the SDM package^12^. The variables used were 25 initially but trimmed after multicollinearity was detected. Multicollinearity was checked using the VIF procedure of the USDM package in the R software program^13^. Variables that have more than 0.7 correlation coefficients were removed from the dataset. Accordingly, 14 variables were removed, and the remaining 11 were used to develop the model.

### Model development

The model was developed in R software^14^. The biomod2 package^15^ with the Ensemble approach^16^ was used to develop a predictive model that delineates the global suitability distribution of AHS^17,18^. An ensemble modeling comprises ten models to pool results so that the ensemble model will have better performance. The models used for this study were Artificial Neural Networks (ANN), Surface Range Envelope (SRE), Flexible Discriminant Analysis (FDA), General Linear Models (GLM), General Additive Models (GAM), General Boosted Models (GBM), Classification Tree Analysis (CTA), Multiple Adaptive Regression Splines (MARS), Random Forests (RF), and Maximum Entropy model^19^. As these models require absence and present records, a dataset of 10,000-pseudo absence was generated with the Surface Range Envelope (SRE) model. Under the SRE strategy, absence points are selected in the area by the models, which are dissimilar from the presence points of species. The areas where the species were not recorded and where the environmental conditions cause potential absence^20^.

Modeling options in Biomod2 were set to default and the algorithm runs threefold with a total of 30 outputs for the ten models. Data were split into training and evaluation sets. 80% of the data was used to develop the model, while 20% was used to evaluate its performance. Area Under the Receiver Operating Curve (AUC) ROC curve, Kappa and the true skill statistic (TSS) were used to evaluate the model performance. TSS value of more than 0.8 was used to ensemble the 30 outputs of the ten models.

Global Suitability level was generated with mean probability, weighted mean (wm) and committee averaging (CA) values. Committee averaging has a dual purpose in ensemble modeling. Firstly, it can be used to predict suitable niches and secondly, it can also be used to evaluate the model's performance^21,22^. The current suitability distribution result was further projected to predict the disease's future distribution gradient under a moderate global warming scenario of the Representative Concentration Pathway (RCP) 4.5 from 2020 to 2060. RCP 4.5 is described by the IPCC as an intermediate scenario. Emissions in RCP 4.5 peak around 2040 then decline^23^. It is a stabilization scenario and thus assumes the imposition of emissions mitigation policies. Among the common RCPs, (RCP2.6, RCP4.5, RCP6, and RCP8.5), RCP 4.5 assumes CO2 emission will be managed in the near future. Unlike RCP 2.6 (the lowest scenario) and RCP 8.5 (the extreme scenario), RCP 4.5 is a reasonably balanced and likely assumption which is why we opt to use it to predict future scenarios of AHS outbreaks.

All the suitability map outputs generated by R software^14^ were converted to a Geo-Tiff raster format, styled and prepared in the QGIS software program^24^. The suitability level in CA, mean, and weighted mean ranged from 10, 250, 500, 750 and nearly 1000 when classified in continuous linear values in the QGIS software program. These values were divided by 1000. Hence the suitability values were approximated to zero (unsuitable), 0.25 (moderately suitable), 0.5 (considerably suitable), 0.75 (suitable) and nearly 1(highly suitable).

## Results

### Individual model evaluation and variable importance

Most of the individual models performed very well in all evaluation metrics employed. By TSS evaluation, RF, GBM, GLM, GAM perform well, with values of 0.98, 0.96, 0.96, 0.94, respectively. Similarly, with ROC evaluation metrics, the models mentioned above outperformed the rest of the models. SRE and Maxent performed less from all ten models with a TSS value of 0.67 and 0.77, respectively (Table 1).

**Table 1:** Individual model performance by ROC, TSS and Kappa evaluation metrics.

Evaluation	RF	GAM	GLM	GBM	CTA	ANN	MARS	SRE	FDA	Maxent
TSS	0.99	0.94	0.96	0.96	0.89	0.86	0.95	0.68	0.86	0.77
ROC	0.99	0.97	0.98	0.99	0.94	0.95	0.99	0.84	0.96	0.89
Kappa	0.92	0.76	0.54	0.84	0.43	0.35	0.64	0.51	0.47	0.65

**Table 2:** The ensemble model performance by Kappa, TSS, and ROC evaluation metrics.

Evaluation metrics	Mean suitability	CA	Weighted mean
KAPPA	0.95	0.95	0.95
TSS	0.99	0.97	0.98
ROC	1.0	0.99	1.0

CA, committee averaging.

Variance plot of ROC versus TSS values for each model indicated that TSS values had higher variability than ROC (supplementary file Fig. 2). Response curves for some selected models have been depicted in (supplementary file Fig. 3). As far as the variable contribution is concerned, in RF, altitude and solar radiation contributed the highest share. In a few models, altitude and temperature variables contributed the highest share, while precipitation variables had the highest contribution in other models. However, solar radiation alone contributed nearly a quarter of the contribution in almost all models (supplementary file Table 2).

**Figure 1:**
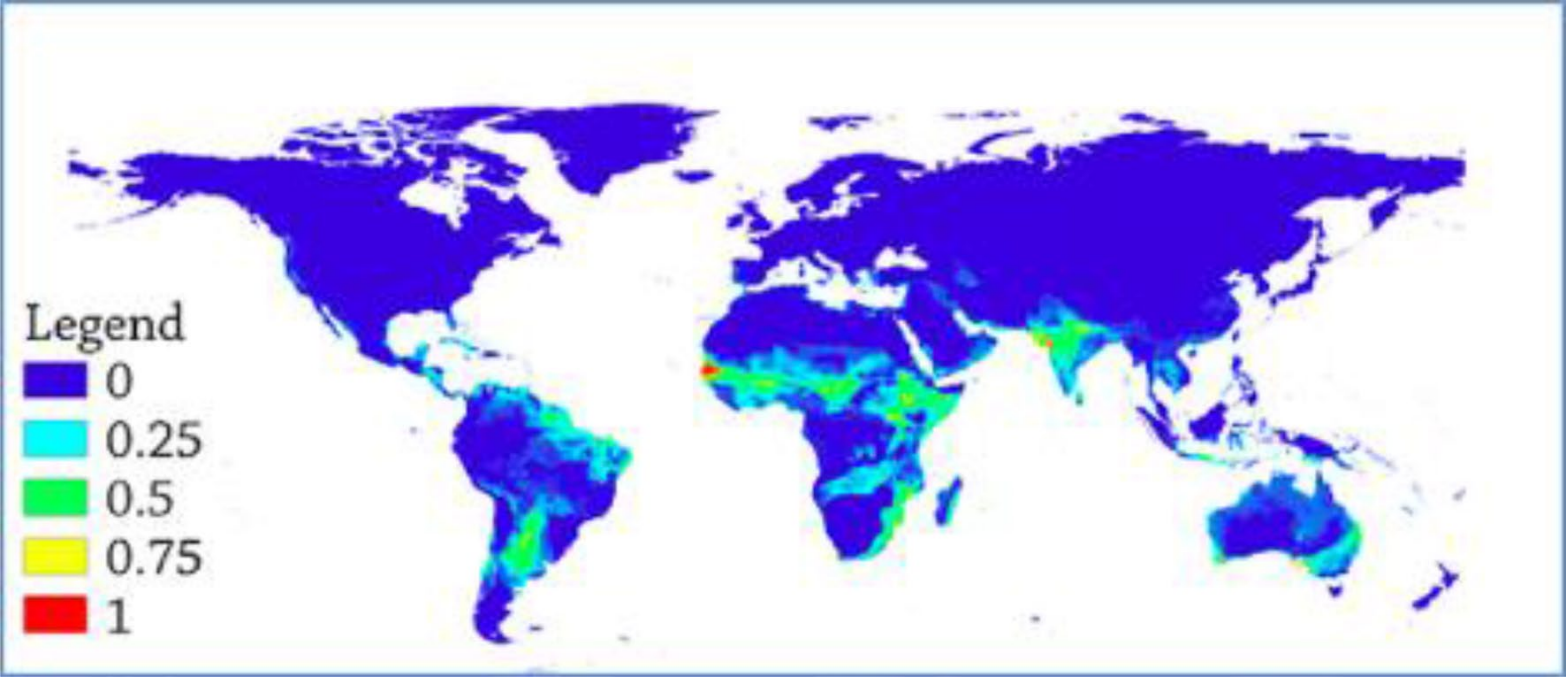
Mean global suitability depiction of AHSv. (The warmer colors depict highly suitable territories while cooler colors depict non suitable locations).

**Figure 2:**
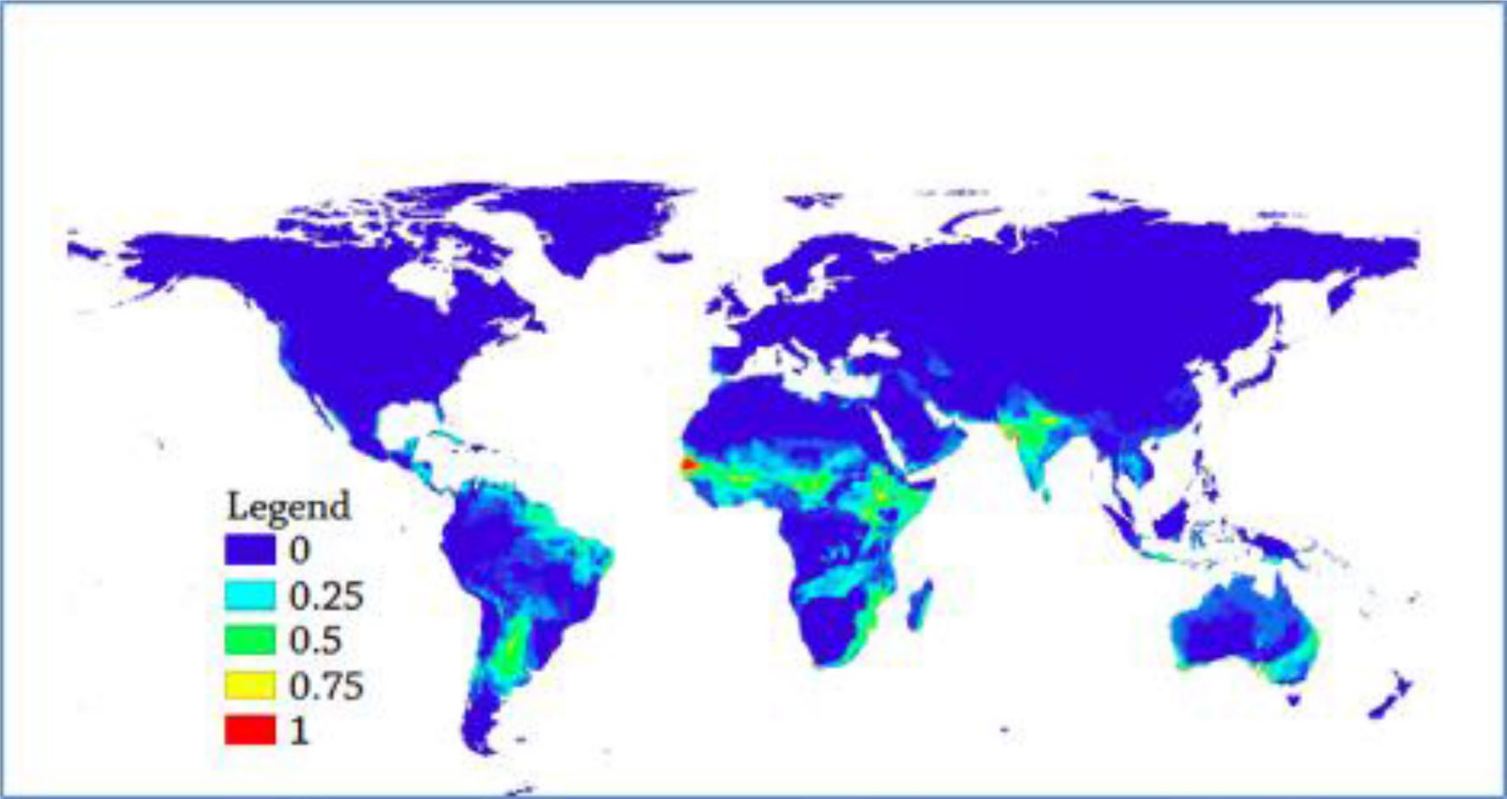
Weighted Mean global suitability depiction of AHS. (The warmer colors depict highly suitable territories while cooler colors depict non suitable locations).

**Figure 3:**
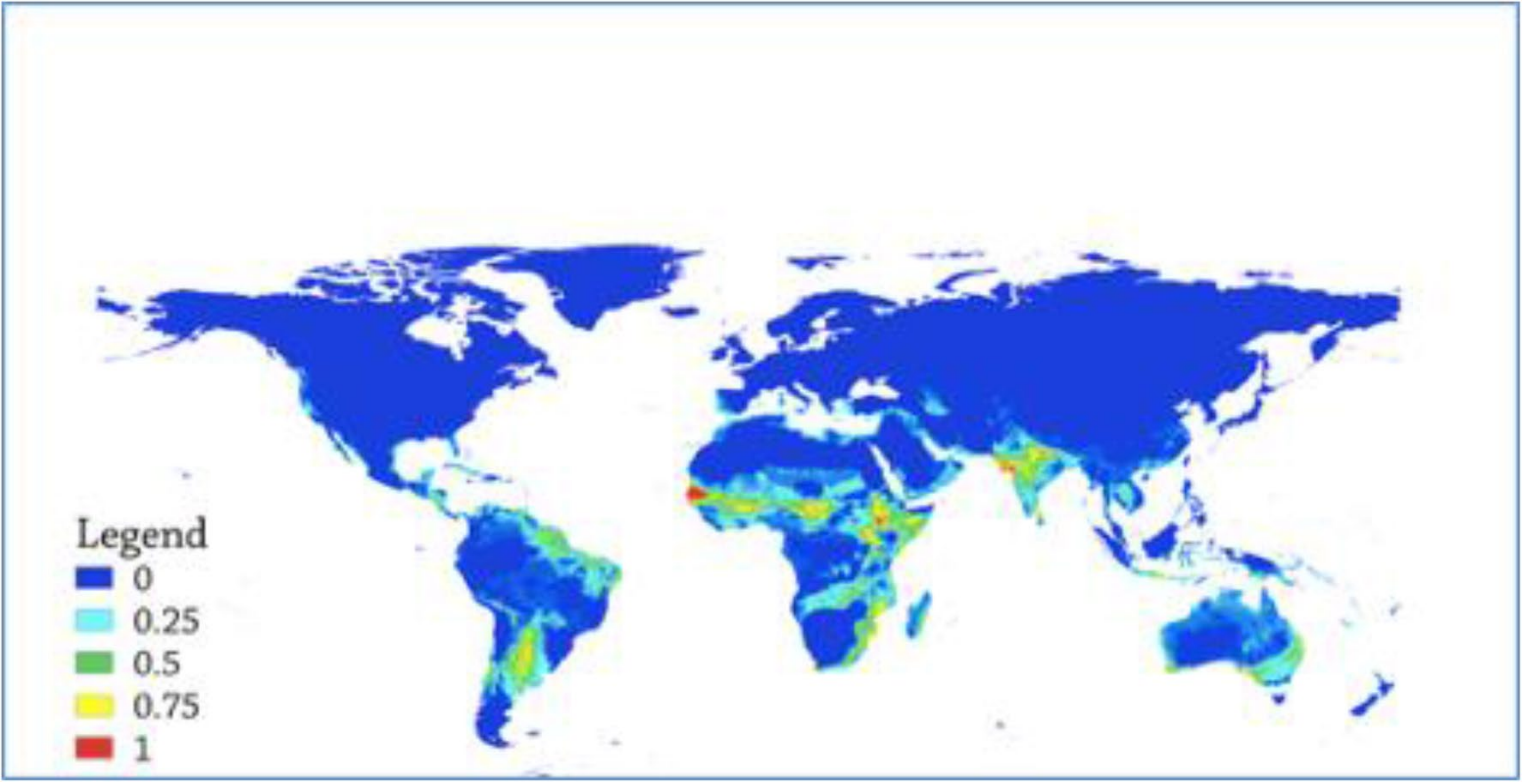
Committee averaging of the ensemble model depicting both suitability level and model uncertainty.

### Ensemble model

#### Model performance

With all evaluation methods used, the ensemble model has better performance than individual models. For example, by ROC evaluation, the model had a perfect performance with a value of 1.0, followed by TSS (0.99) and Kappa (0.95) (Table 2).

#### Variable contribution in the ensemble model

In line with individual models, temperature variables contributed the highest share in the ensemble model. Solar radiation alone contributed 36.83%, while the average maximum temperature had a 17.1% contribution. Similarly, average precipitation of the year (14.34%), altitude (7.61%), and precipitation seasonality (6.41%) also contributed a significant share (supplementary file Table 3).

### Predicted suitable territories of the world for AHSv

The suitability level for the virus was generated in the ensemble modeling with mean suitability level (Fig. 1), committee averaging (Fig. 2) and weighted mean suitability levels (Fig. 3). The model indicates that the sub-Sahara African continent is the most suitable area for the virus. Senegal, Burkina Faso, Niger, Nigeria, Ethiopia, Sudan, Somalia, South Africa, Zimbabwe, Madagascar and Malawi are African countries identified as suitable niches for the virus. Besides, the Middle East, India, Australia, and most of South America (Brazil, Paraguay and Bolivia) have been found suitable territories for the virus (Fig. 1).

### Model uncertainty

**Figure 4:**
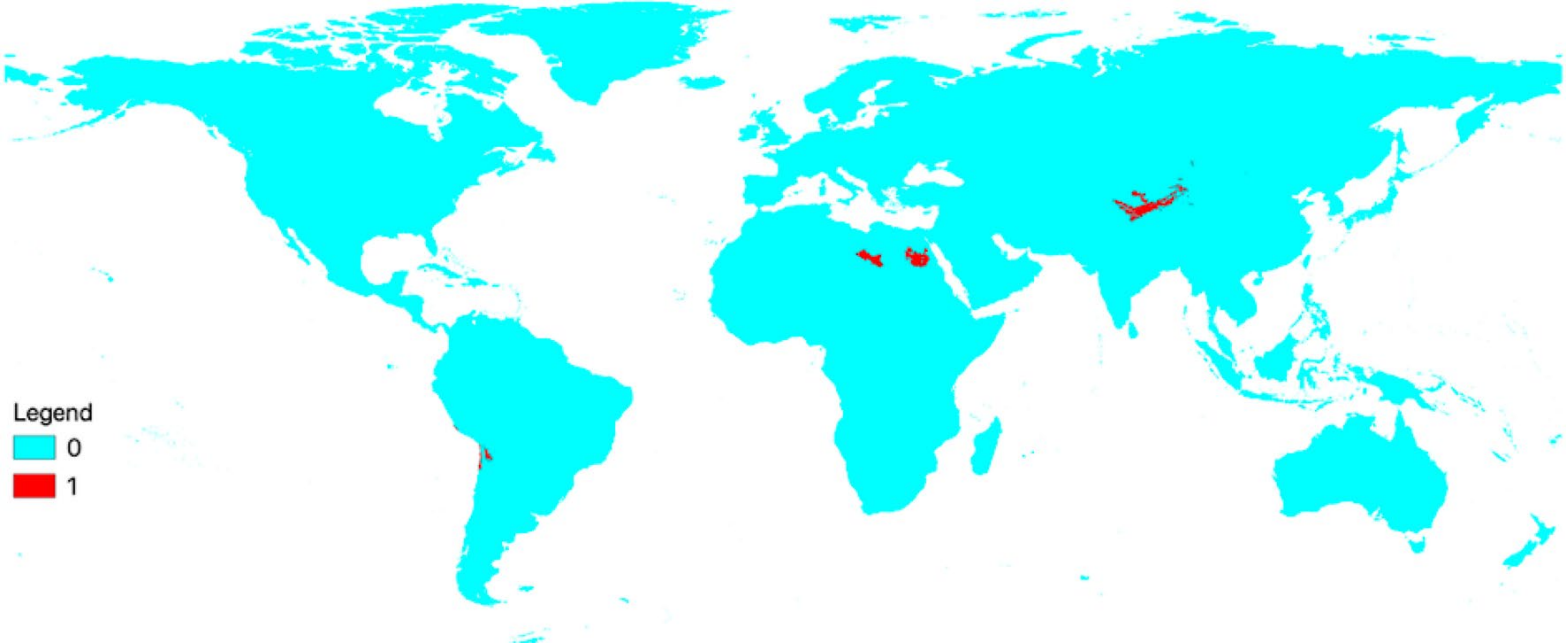
Model uncertainty measurement with a clamping mask value. The warmer color indicates areas where the model was uncertain, while blue colors depict the model's prediction was certain.

Model uncertainty was measured model’s by clamping mask value. Expressed uncertainties of the model were in North America, Russia, and South America (Fig. 4).

### Predicted Future suitability distribution of AHSv

**Figure 5:**
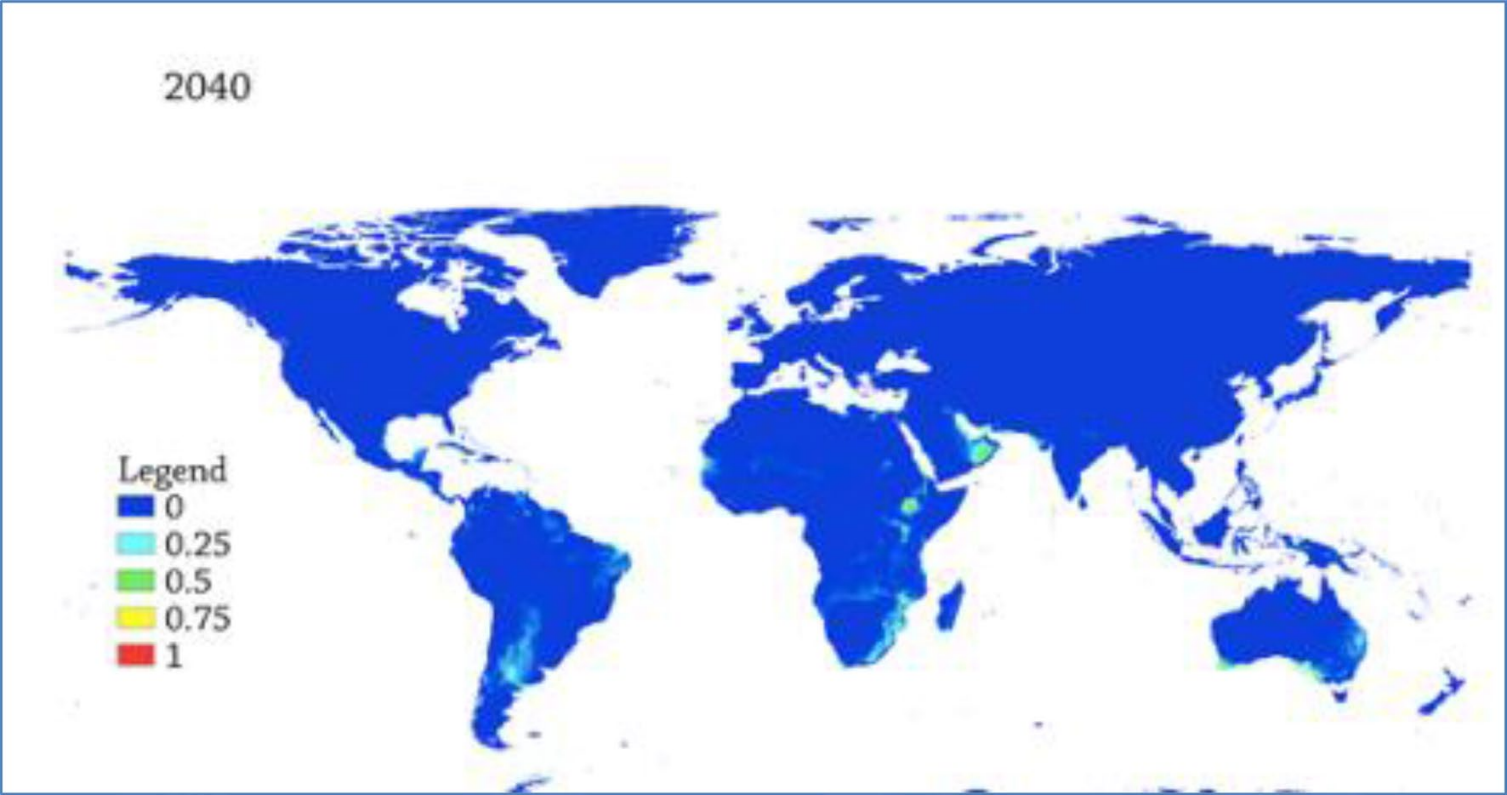
Predicted future global distribution gradient of AHS from 2020 to 2040. The warmer areas depict suitable areas while the cooler colors depict unsuitable localities.

**Figure 6:**
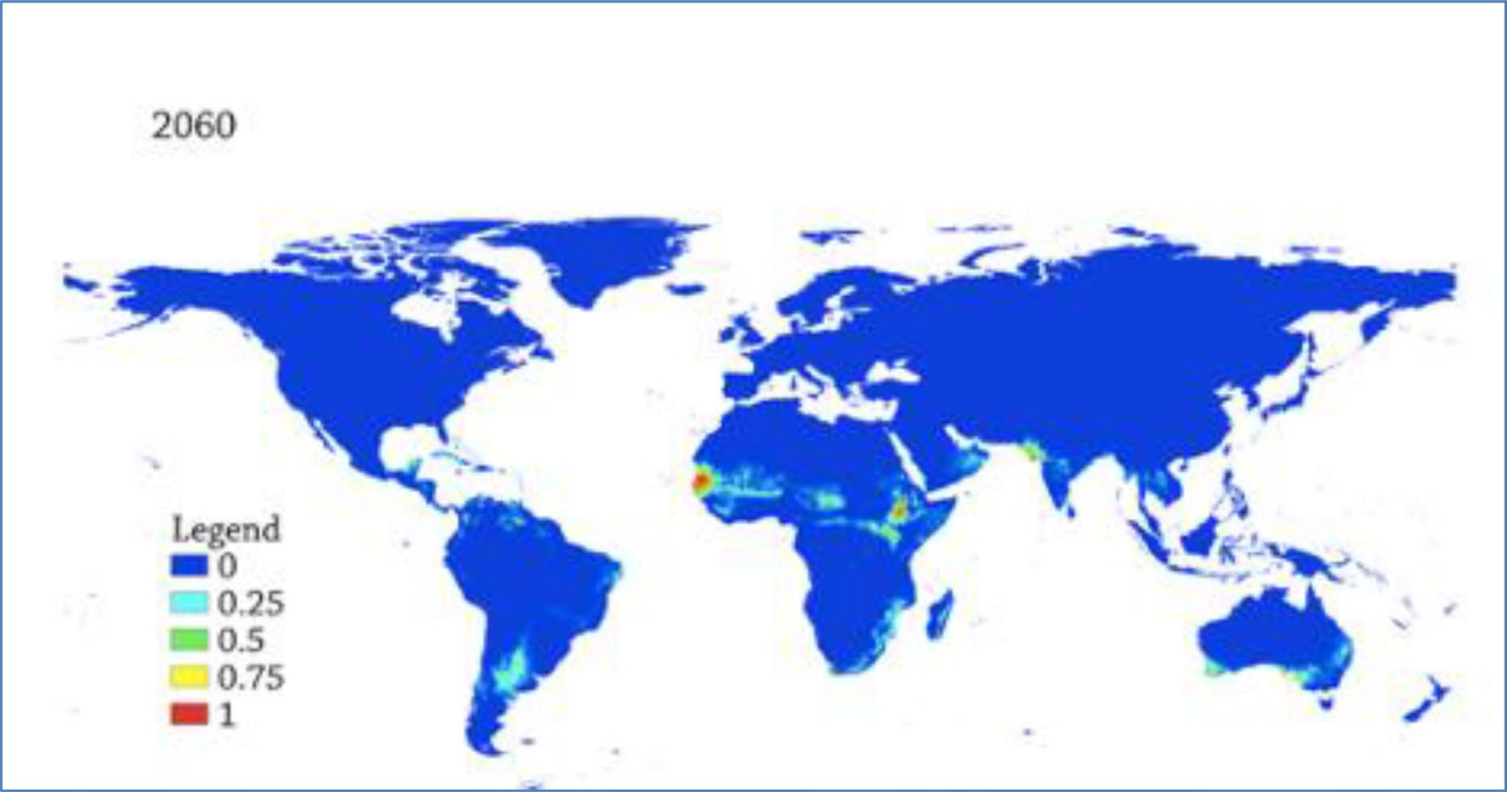
Predicted future global distribution gradient of AHS in from 2040 to 2060. The warmer colors depict suitable areas while the cooler colors depict unsuitable localities.

The distribution of suitable niches was projected to the years 2020 to 2040 and2040 to 2060. The results indicate that the suitability level will diminish between 2020 and 2040 than the current suitability level (Fig. 5). However, from 2040 to 2060, the suitable niches will grow wider than 2020 to 2040 but smaller than the current suitability level (Fig 6).

## Discussion

African horse sickness is one of the devastating health threats to the equid family. Mostly donkeys, mule and zebra are believed to be reservoirs; they are less susceptible to the disease^2^. So far, efforts have been made to develop effective prevention and control approaches. These control approaches are focused on three components, namely, quarantine, vector control and vaccination. In support of these approaches, vaccine development efforts have been implemented to develop effective vaccines successfully. Besides, vector control methods primarily focused on the confinement of animals in the active season of vectors have been practiced.

This is the first of its kind attempt to model the suitability niche for AHSv from retrospective outbreak records to the best of our knowledge. The model comes in line with previous knowledge of the disease distribution territories known for its occurrence. The ecological niche model trained with the ensemble modeling had perfect accuracy (1.0) with ROC evaluation metrics. Besides, it had 0.99 with TSS and 0.95 in Kappa accuracy evaluation, indicating correctly classifying suitable and unsuitable territories of the world for AHSv. The ensemble model's accuracy metrics were better than individual models indicating the ensemble approach was the best choice than using individual models alone^25–27^. Bioclimatic variables like Solar radiation, mean maximum temperature, mean precipitation of the year, altitude and precipitation seasonality contributed 36.83%, 17.1%, 14.34%, 7.61%, and 6.4%, respectively. These variables alone account for more than 80% share of the model. Temperature variables are known to determine the survival of vectors responsible for disease transmission. The tropical area had moderately hot weather conditions coupled with higher precipitation to facilitate rain, increasing humidity levels; it is ideal for insects' reproduction. Furthermore, suitable niche identification to identify suitable territories for Culicoides has been delineated^4,28,29^. Even though these ENMs are outstanding works identifying favorable territories, they fail to incorporate vectors other than the Culicoides genera. Other species involved as a vector for AHSv transmission have been reported recently^1,30–33^. Some include Mosquitoes of the Aedes, Culex and Anopheles genera, ticks (Hyalomma and Rhipicephalus)^2^.

African countries like Senegal, Burkina Faso, Niger, Nigeria, Ethiopia, Sudan, Somalia, South Africa, Zimbabwe, Madagascar and Malawi are identified as suitable niches for the virus. Humid sub-Saharan Africa is very suitable for any disease vectors due to prolonged rain with favorable temperatures^34–36^. These variables, coupled with the presence of susceptible horses and maintaining zebras, accelerate outbreaks to be endemic and, in some cases, hyperendemic outbreaks in the African continent. Furthermore, the Middle East (Yemen, Oman, Saudi Arabia and territories) were found marginally suitable for the virus. As these areas are known to harbor a large population of Arabian horses, they can be primarily affected by the disease. Besides, disease-free areas were also depicted as suitable in this model, indicating the need for the strict prohibition of introducing positive animals in these countries can result in maintained disease cases for a prolonged period as these areas are suitable for disease, i.e., suitable for any possible vectors capable of transmitting the virus, primarily midges of the Culicoides genera.

OIE lists disease-free countries like India, Australia, and the south Americas (Brazil, Paraguay and Bolivia) were found suitable areas for the virus. The India southern borders were highly suitable for the virus due to suitable bioclimatic conditions for the thriving of the vectors. Furthermore, most of Australia and brazil were also suitable for the virus, which can be due to suitable weather conditions similar to African countries in terms of temperature and humidity. Even though some territories are free, yet their territories were suitable for the diseases. If the virus reaches these territories and is maintained in any possible vector, the virus can persist for prolonged periods affecting the equine population.

Future projections from 2020 to 2040 and 2040 to 2060 indicated that the suitable territories would diminish than the current distribution gradient. As reasonable global warming will be imminent in the coming years, this argument seems unlikely to decrease infectious diseases like AHSv. However, the possible reason can be that temperature will increase, affecting the dynamics of vectors. Besides, for an outbreak to occur, moderate temperature along with higher precipitation rates should exist. However, in the future, the conditions may not exist as rainfall will decrease. In contrast, the temperature rises scenarios favor the vector dynamics negatively, directly diminishing vector-borne infectious diseases.

The model was magnificent in every evaluation metric employed and depicted suitable territories previously known with the disease to occur. However, it had its limitations. Among them, the inability to incorporate outbreak occurrence data other than African countries and predict for wider area are prominent. ENMs for wider areas come with under or over estimation of suitability level. Besides, background pseudo absence sampling from non-occurrence locations using SRE may result in inaccurate metrics. Due to these reasons, we advise readers to consider these limitations whenever they want to use this model.

## Conclusions

The model is the first to use ENM with bioclimatic risk factor identification for AHSv outbreaks worldwide**.** It had a perfect classification capability of suitable and unsuitable niches of the world. Bioclimatic variables like solar radiation, maximum temperature, and precipitation variables contributed to the model's highest share. Endemic territories of Sub-Saharan Africa and the Arabian countries were found highly suitable. Furthermore, OIE's disease-free areas, like India, Australia, and Brazil, were found suitable for the disease. We believe this model can be used as an epidemiological tool in planning control and surveillance against diseases nationally or internationally.

## Supplementary Information

Supplementary Information 1.

Supplementary Information 2.

Supplementary Information 3.
