# Supplementary material for: Ecological niche modeling predicting the potential distribution of African horse sickness virus from 2020 to 2060
Source: Sci Rep. 2022 Feb 2;12:1748. doi: 10.1038/s41598-022-05826-3 (PMC8811056; doi:10.1038/s41598-022-05826-3)
Supplement: Supplementary file 1 — Supplementary Information 1. [file 41598_2022_5826_MOESM1_ESM.docx]

**Table 1.** Bioclimatic and topographic variables used in the ensemble model.

| **Variable Code** | **Variable Name** | **Source** |
| --- | --- | --- |
| bio_2 | Mean Diurnal Range | WorldClim 2.1 |
| bio_14 | Mean Precipitation of Driest Month(mm) | WorldClim 2.1 |
| bio_15 | Precipitation Seasonality (CV) | WorldClim 2.1 |
| Tmax | Mean Annual Maximum Temperature (^o^c) | WorldClim 2.1 |
| Tmin | Mean Annual Minimum Temperature (^o^c) | WorldClim 2.1 |
| bio_18 | Mean Precipitation of Warmest Quarter(mm) | WorldClim 2.1 |
| bio_19 | Mean Precipitation of Coldest Quarter (mm) | WorldClim 2.1 |
| Prec | Mean Annual Precipitation (mm) | WorldClim 2.1 |
| Srad | Solar radiation (kJ /day) | WorldClim 2.1 |
| wc2.1_2.5m_elev | Elevation/Altitude (m) | WorldClim 2.1 |
| Wind | Wind Speed (m/s) | WorldClim 2.1 |

**Table 2.** Variable contribution to the Global suitability distribution of AHS in the individual models.

| **Variable Name** | RF | GAM | GLM | GBM | CTA | ANN | MARS | SRE | FDA | Maxent | Mean |
| --- | --- | --- | --- | --- | --- | --- | --- | --- | --- | --- | --- |
| Mean Diurnal Range | 5.37 | 8.28 | 2.53 | 13.06 | 13.03 | 7.2 | 4 | 14.37 | 0.95 | 10.11 | 7.9 |
| Precipitation of Driest Month | 1.74 | 4.18 | 8.23 | 0.26 | 0.85 | 8.82 | 2.53 | 1.29 | 0 | 2.97 | 3.1 |
| Precipitation Seasonality (CV) | 10.58 | 12.27 | 11.68 | 7.92 | 3.44 | 2.3 | 10.45 | 6.87 | 2.56 | 11.49 | 8.0 |
| Annual Maximum Temperature | 4.74 | 14.52 | 17.13 | 14.13 | 0 | 22.84 | 30.28 | 15.11 | 3.86 | 6.64 | 12.9 |
| Annual Minimum Temperature | 6.95 | 15.19 | 16.54 | 1.15 | 2 | 8.76 | 12.97 | 15.99 | 11.14 | 6.99 | 9.8 |
| Precipitation of Warmest Quarter | 5.37 | 2.48 | 0.91 | 0.08 | 0 | 8.76 | 0 | 7.59 | 5.23 | 7.7 | 3.8 |
| Precipitation of Coldest Quarter | 12.8 | 4.68 | 9.17 | 0.69 | 0 | 11.15 | 1.43 | 2.69 | 3.9 | 10.5 | 5.7 |
| Precipitation | 7.9 | 9.36 | 8.33 | 17.71 | 27.21 | 6.61 | 7.92 | 7.99 | 17.6 | 11.02 | 12.2 |
| Solar radiation | 17.85 | 15.62 | 17.43 | 42.61 | 48.63 | 7.13 | 19.6 | 20.74 | 50.96 | 15.22 | 25.6 |
| Elevation/Altitude | 26.22 | 9.51 | 4.13 | 2.37 | 4.82 | 13.72 | 10.81 | 4.57 | 3.29 | 12.7 | 9.2 |
| Wind Speed | 0.47 | 3.9 | 3.93 | 0.04 | 0 | 2.69 | 0 | 2.8 | 0.54 | 4.64 | 1.9 |

**Table 3.** Variable contribution in the ensemble model.

| **Variables** | **% Contribution (mean)** | **% Contribution**  **(CA)** | **% Contribution (WM)** |
| --- | --- | --- | --- |
| Mean Diurnal Range | 3.60 | 3.27 | 3.73 |
| Precipitation of Driest Month | 2.63 | 2.42 | 2.81 |
| Precipitation Seasonality (CV) | 6.41 | 5.62 | 6.88 |
| Annual Maximum Temperature | 17.10 | 16.03 | 17.12 |
| Annual Minimum Temperature | 5.02 | 5.72 | 5.34 |
| Precipitation of Warmest Quarter | 0.87 | 2.00 | 0.80 |
| Precipitation of Coldest Quarter | 4.70 | 5.59 | 4.83 |
| Precipitation | 14.34 | 16.13 | 13.97 |
| Solar radiation | 36.83 | 35.31 | 36.32 |
| Elevation/Altitude | 7.61 | 6.96 | 7.16 |
| Wind Speed | 0.89 | 0.96 | 1.05 |
