# Supplementary material for: Ecological niche modeling predicting the potential distribution of African horse sickness virus from 2020 to 2060
Source: Sci Rep. 2022 Feb 2;12:1748. doi: 10.1038/s41598-022-05826-3 (PMC8811056; doi:10.1038/s41598-022-05826-3)
Supplement: Supplementary file 2 — Supplementary Information 2. [file 41598_2022_5826_MOESM2_ESM.docx]

**
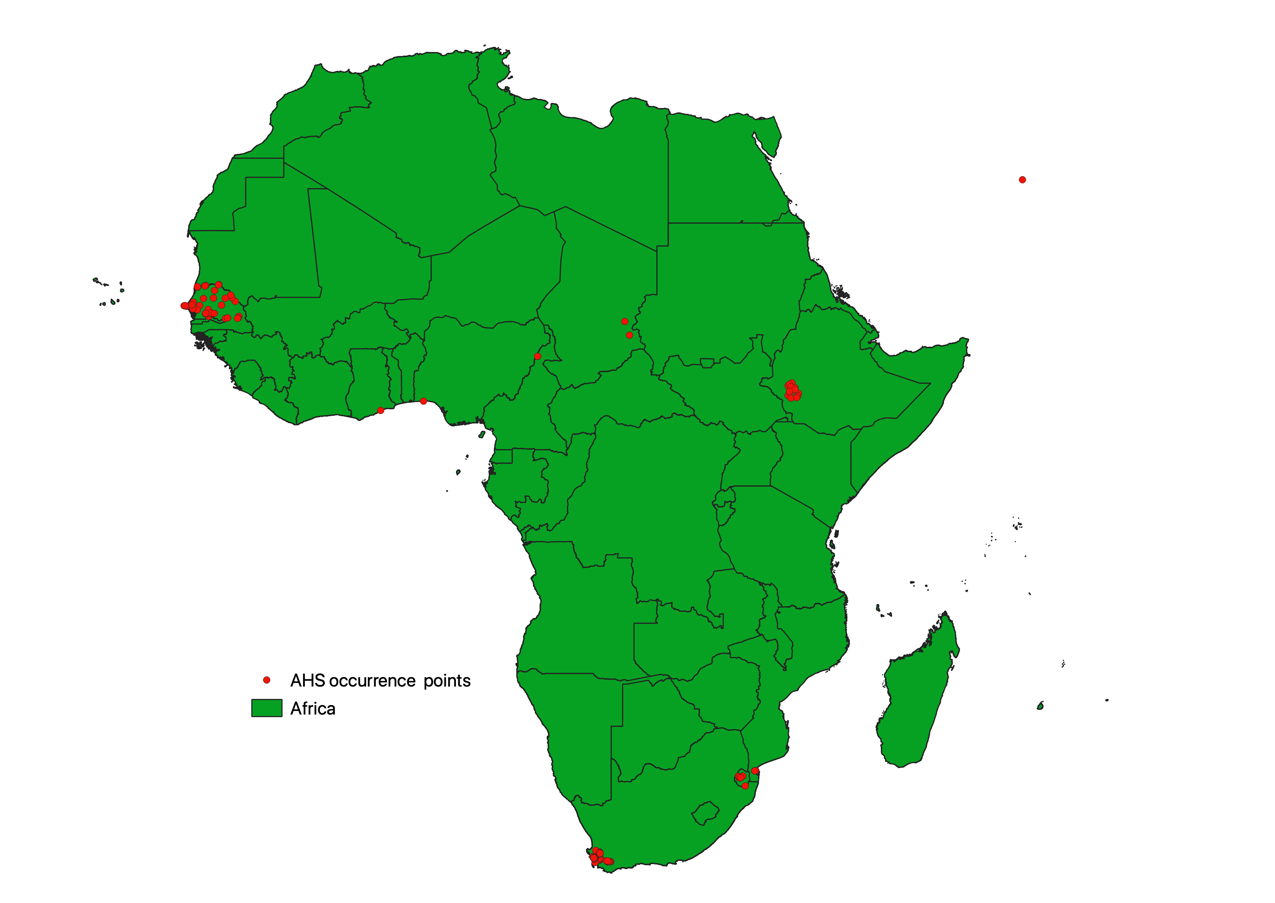
**

**Fig 1. AHS occurrence points used to develop the model (the map was generated using QGIS software version 3.18)**

**
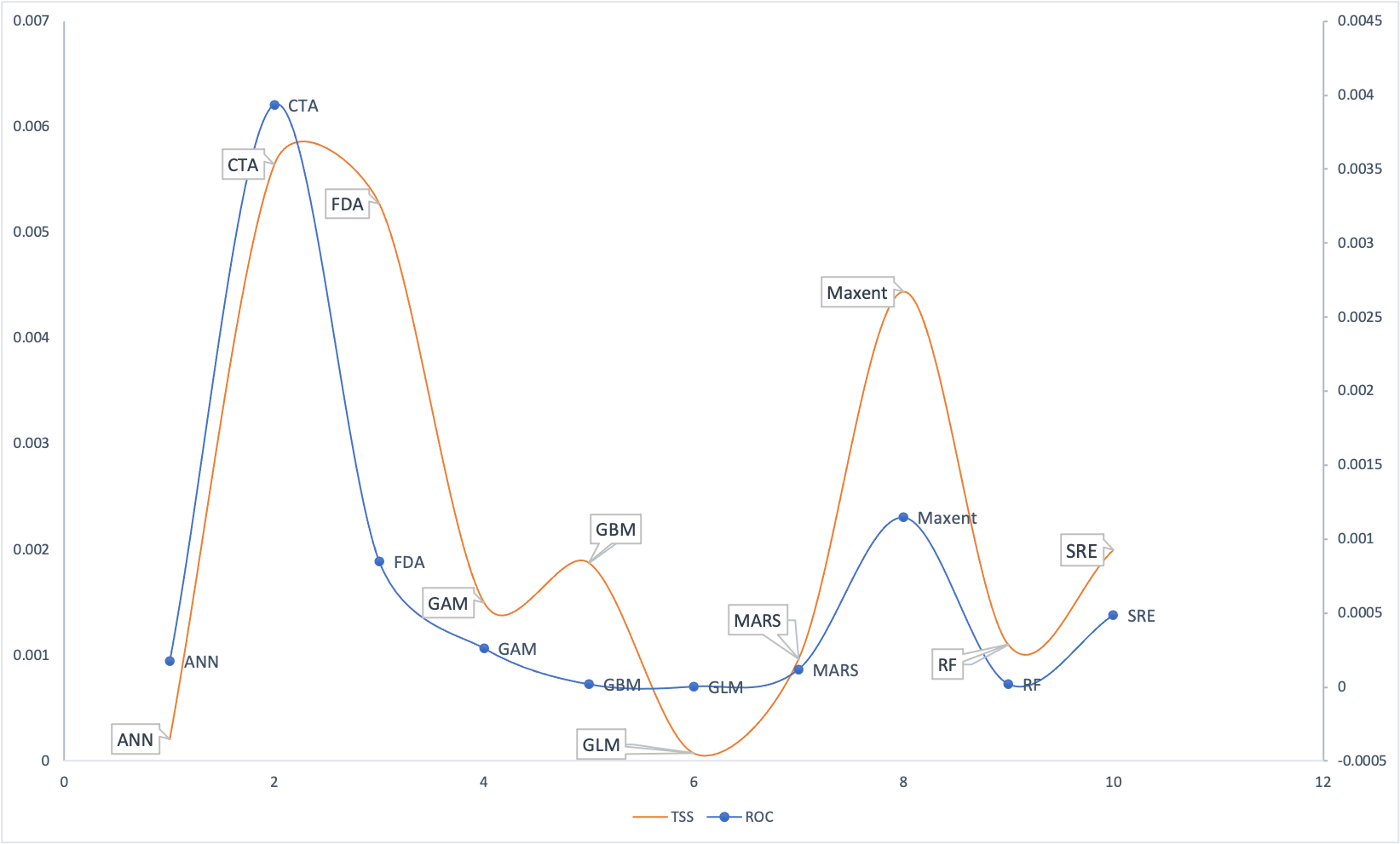
**

**Fig 2.** Variance plot of Roc and TSS values of each model used to develop the ensemble model

**
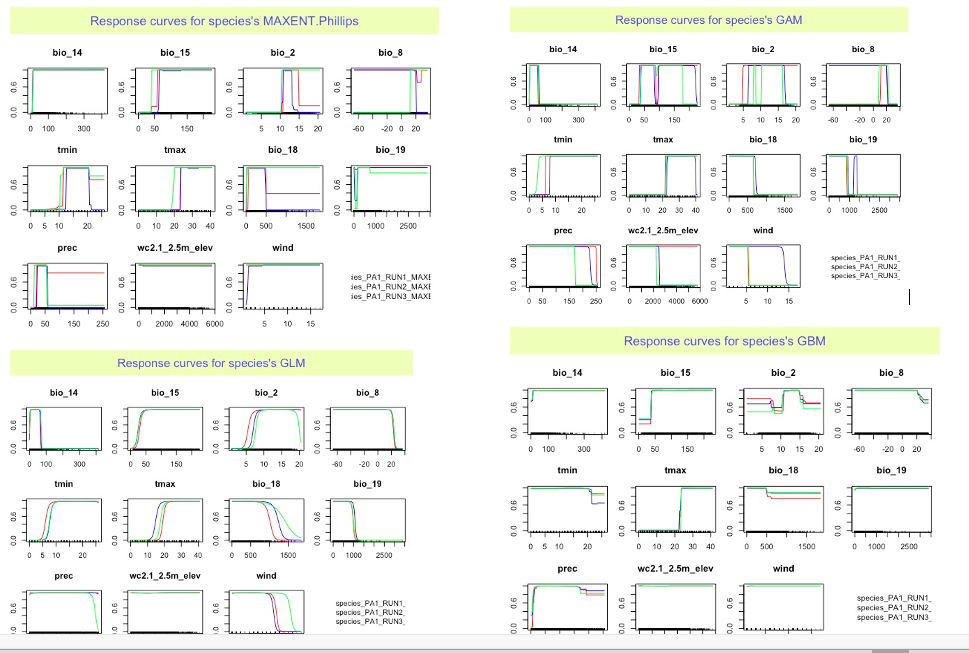
**

**Fig 3.** Response curves of some of the models used (Maxent top left, GAM top right, GLM bottom left, and GBM bottom right)
