# Supplementary material for: Ecological niche modeling predicting the potential distribution of African horse sickness virus from 2020 to 2060
Source: Sci Rep. 2022 Feb 2;12:1748. doi: 10.1038/s41598-022-05826-3 (PMC8811056; doi:10.1038/s41598-022-05826-3)
Supplement: Supplementary file 3 — Supplementary Information 3. [file 41598_2022_5826_MOESM3_ESM.docx]

**Georeferenced African horse sickness outbreak occurrence points**

| Disease | Latitude | Longitude | country |
| --- | --- | --- | --- |
| African horse sickness | -26.513987 | 31.269146 | Swaziland |
| African horse sickness | 12.196 | 21.6057 | Chad |
| African horse sickness | 13.3907 | 21.1892 | Chad |
| African horse sickness | 10.339595 | 13.562534 | Cameroon |
| African horse sickness | 10.339344 | 13.561828 | Cameroon |
| African horse sickness | -26.467 | 31.389 | Swaziland |
| African horse sickness | -26.485693 | 31.191971 | Swaziland |
| African horse sickness | -26.519201 | 31.214828 | Swaziland |
| African horse sickness | -26.3562 | 31.52398 | Swaziland |
| African horse sickness | -26.485693 | 31.191971 | Swaziland |
| African horse sickness | -26.485693 | 31.191971 | Swaziland |
| African horse sickness | -26.499 | 31.344 | Swaziland |
| African horse sickness | -26.519201 | 31.214828 | Swaziland |
| African horse sickness | -25.87642 | 32.6626 | Mozambique |
| African horse sickness | -33.6 | 18.9 | South Africa |
| African horse sickness | -33.6 | 19 | South Africa |
| African horse sickness | -33.6 | 19 | South Africa |
| African horse sickness | -33.6 | 18.8 | South Africa |
| African horse sickness | -33.5 | 18.9 | South Africa |
| African horse sickness | -33.6 | 18.9 | South Africa |
| African horse sickness | -33.7 | 19 | South Africa |
| African horse sickness | -33.7 | 19 | South Africa |
| African horse sickness | -25.9058 | 32.5812 | Mozambique |
| African horse sickness | -33.7781 | 19.6791 | South Africa |
| African horse sickness | -33.8462 | 19.96263 | South Africa |
| African horse sickness | -33.83319 | 19.7636 | South Africa |
| African horse sickness | -33.8397 | 19.92478 | South Africa |
| African horse sickness | -33.86184 | 19.70181 | South Africa |
| African horse sickness | -33.81348 | 19.78239 | South Africa |
| African horse sickness | -33.84211 | 19.83086 | South Africa |
| African horse sickness | -26.364 | 31.124 | Swaziland |
| African horse sickness | -26.419 | 31.214 | Swaziland |
| African horse sickness | -33.83858 | 19.73688 | South Africa |
| African horse sickness | -33.67511 | 18.94196 | South Africa |
| African horse sickness | -33.015 | 18.997 | South Africa |
| African horse sickness | -33.57414 | 19.07234 | South Africa |
| African horse sickness | -32.86 | 18.66 | South Africa |
| African horse sickness | -33.181 | 19.01 | South Africa |
| African horse sickness | -33.842111 | 19.8308611 | South Africa |
| African horse sickness | -25.892423 | 32.557787 | Mozambique |
| African horse sickness | -25.9058 | 32.5812 | Mozambique |
| African horse sickness | -33.181 | 19.01 | South Africa |
| African horse sickness | -33.00692 | 18.99967 | South Africa |
| African horse sickness | -33.56897 | 19.05458 | South Africa |
| African horse sickness | -33.18102 | 19.01002 | South Africa |
| African horse sickness | -33.181 | 19.01 | South Africa |
| African horse sickness | -33.565 | 18.938 | South Africa |
| African horse sickness | -33.16764 | 19.02366 | South Africa |
| African horse sickness | -33.17845 | 19.01073 | South Africa |
| African horse sickness | -33.181 | 19.01 | South Africa |
| African horse sickness | -33.181 | 19.01 | South Africa |
| African horse sickness | -33.181 | 19.01 | South Africa |
| African horse sickness | -33.1786 | 18.9952 | South Africa |
| African horse sickness | -33.181 | 19.01 | South Africa |
| African horse sickness | -33.1775 | 19.0077 | South Africa |
| African horse sickness | -33.181 | 19.01 | South Africa |
| African horse sickness | -33.1854 | 18.9498 | South Africa |
| African horse sickness | -33.181 | 19.01 | South Africa |
| African horse sickness | -33.0219 | 18.9963 | South Africa |
| African horse sickness | -33.1866 | 18.9933 | South Africa |
| African horse sickness | -33.181 | 19.01 | South Africa |
| African horse sickness | -33.0174 | 19.0084 | South Africa |
| African horse sickness | -33.048 | 18.9652 | South Africa |
| African horse sickness | -33.111 | 19.02406 | South Africa |
| African horse sickness | -33.1115 | 19.02406 | South Africa |
| African horse sickness | -33.914774 | 18.582703 | South Africa |
| African horse sickness | -26.499 | 31.344 | Swaziland |
| African horse sickness | -33.46481 | 18.47264 | South Africa |
| African horse sickness | -33.50589 | 18.44868 | South Africa |
| African horse sickness | -33.52039 | 18.59587 | South Africa |
| African horse sickness | -33.50589 | 18.44868 | South Africa |
| African horse sickness | -33.53989 | 18.52347 | South Africa |
| African horse sickness | -33.50596 | 18.44884 | South Africa |
| African horse sickness | -33.53703 | 18.52724 | South Africa |
| African horse sickness | -33.50774 | 18.45011 | South Africa |
| African horse sickness | -33.61694 | 18.52919 | South Africa |
| African horse sickness | -33.50576 | 18.44414 | South Africa |
| African horse sickness | -33.4965 | 18.44903 | South Africa |
| African horse sickness | -33.54067 | 18.52236 | South Africa |
| African horse sickness | -33.57973 | 18.57947 | South Africa |
| African horse sickness | -33.50406 | 18.44343 | South Africa |
| African horse sickness | -33.396 | 18.395 | South Africa |
| African horse sickness | -33.52461 | 18.47303 | South Africa |
| African horse sickness | -33.54099 | 18.52148 | South Africa |
| African horse sickness | -33.50539 | 18.44894 | South Africa |
| African horse sickness | -33.5095 | 18.50312 | South Africa |
| African horse sickness | -33.51062 | 18.47576 | South Africa |
| African horse sickness | -33.4865 | 18.48886 | South Africa |
| African horse sickness | -33.51978 | 18.47997 | South Africa |
| African horse sickness | -33.4865 | 18.4888 | South Africa |
| African horse sickness | -33.48894 | 18.48699 | South Africa |
| African horse sickness | -33.48636 | 18.4784 | South Africa |
| African horse sickness | -33.51978 | 18.47997 | South Africa |
| African horse sickness | -33.51122 | 18.47792 | South Africa |
| African horse sickness | -33.50739 | 18.4515 | South Africa |
| African horse sickness | -33.45893 | 18.48732 | South Africa |
| African horse sickness | -33.50739 | 18.4515 | South Africa |
| African horse sickness | -33.4865 | 18.48886 | South Africa |
| African horse sickness | -33.48894 | 18.48699 | South Africa |
| African horse sickness | -33.55119 | 18.49231 | South Africa |
| African horse sickness | -33.48894 | 18.48699 | South Africa |
| African horse sickness | -33.4865 | 18.48886 | South Africa |
| African horse sickness | -33.55119 | 18.49231 | South Africa |
| African horse sickness | -33.50619 | 18.44879 | South Africa |
| African horse sickness | -33.50511 | 18.44814 | South Africa |
| African horse sickness | -33.51114 | 18.469 | South Africa |
| African horse sickness | -33.60518 | 18.51243 | South Africa |
| African horse sickness | -33.5095 | 18.4683 | South Africa |
| African horse sickness | -33.51386 | 18.48383 | South Africa |
| African horse sickness | -33.50739 | 18.4525 | South Africa |
| African horse sickness | -33.51353 | 18.48406 | South Africa |
| African horse sickness | -33.51119 | 18.47558 | South Africa |
| African horse sickness | -26.499 | 31.344 | Swaziland |
| African horse sickness | 14.782582 | -16.029023 | Senegal |
| African horse sickness | 14.244513 | -15.20683 | Senegal |
| African horse sickness | 14.40892 | -16.243189 | Senegal |
| African horse sickness | 14.736135 | -17.229055 | Senegal |
| African horse sickness | 16.092511 | -14.732487 | Senegal |
| African horse sickness | 15.397999 | -15.690158 | Senegal |
| African horse sickness | 14.79854 | -16.882508 | Senegal |
| African horse sickness | 5.61 | -0.18333 | Ghana |
| African horse sickness | 14.1725 | -15.1371 | Senegal |
| African horse sickness | 16.6303 | -14.3471 | Senegal |
| African horse sickness | 16.5425 | -15.4992 | Senegal |
| African horse sickness | 14.0298 | -14.9286 | Senegal |
| African horse sickness | 14.6882 | -16.7391 | Senegal |
| African horse sickness | 14.8857 | -16.6952 | Senegal |
| African horse sickness | 14.0518 | -14.8409 | Senegal |
| African horse sickness | 13.8323 | -15.2359 | Senegal |
| African horse sickness | 14.0737 | -14.9835 | Senegal |
| African horse sickness | 14.9296 | -16.7061 | Senegal |
| African horse sickness | 16.5096 | -15.598 | Senegal |
| African horse sickness | 16.4876 | -15.565 | Senegal |
| African horse sickness | 15.149 | -12.9097 | Senegal |
| African horse sickness | 16.5973 | -14.38 | Senegal |
| African horse sickness | 15.4782 | -13.217 | Senegal |
| African horse sickness | 14.8198 | -14.1167 | Senegal |
| African horse sickness | 15.7086 | -13.3157 | Senegal |
| African horse sickness | 14.0847 | -15.4992 | Senegal |
| African horse sickness | 14.4468 | -15.2798 | Senegal |
| African horse sickness | 14.0737 | -14.8409 | Senegal |
| African horse sickness | 14.8857 | -16.7171 | Senegal |
| African horse sickness | 14.7211 | -17.2109 | Senegal |
| African horse sickness | 15.4233 | -13.7875 | Senegal |
| African horse sickness | 14.0847 | -15.4992 | Senegal |
| African horse sickness | 15.0832 | -16.5855 | Senegal |
| African horse sickness | 14.5016 | -16.6293 | Senegal |
| African horse sickness | 16.3779 | -16.2343 | Senegal |
| African horse sickness | 14.0518 | -15.5102 | Senegal |
| African horse sickness | 13.8433 | -12.5916 | Senegal |
| African horse sickness | 16.4437 | -16.2343 | Senegal |
| African horse sickness | 14.8527 | -16.7061 | Senegal |
| African horse sickness | 13.6458 | -13.7766 | Senegal |
| African horse sickness | 13.6897 | -12.6684 | Senegal |
| African horse sickness | 14.1066 | -15.5211 | Senegal |
| African horse sickness | 6.916675 | 35.488331 | Ethiopia |
| African horse sickness | 7.104272 | 36.390881 | Ethiopia |
| African horse sickness | 6.889778 | 36.331222 | Ethiopia |
| African horse sickness | 7.395253 | 35.663433 | Ethiopia |
| African horse sickness | 7.595183 | 35.911614 | Ethiopia |
| African horse sickness | 8.018767 | 35.829531 | Ethiopia |
| African horse sickness | 7.758644 | 35.488389 | Ethiopia |
| African horse sickness | 14.1286 | -15.0164 | Senegal |
| African horse sickness | 14.1066 | -15.5102 | Senegal |
| African horse sickness | 14.0957 | -14.7531 | Senegal |
| African horse sickness | 15.4343 | -14.8189 | Senegal |
| African horse sickness | 13.7116 | -13.5791 | Senegal |
| African horse sickness | 13.6677 | -12.7122 | Senegal |
| African horse sickness | 14.765 | -17.3974 | Senegal |
| African horse sickness | 14.7869 | -17.3096 | Senegal |
| African horse sickness | 7.900311 | 35.610056 | Ethiopia |
| African horse sickness | 7.120747 | 35.663247 | Ethiopia |
| African horse sickness | 6.698028 | 35.739725 | Ethiopia |
| African horse sickness | 6.733333 | 36.239019 | Ethiopia |
| African horse sickness | 7.608094 | 36.083875 | Ethiopia |
| African horse sickness | 7.674653 | 35.728842 | Ethiopia |
| African horse sickness | 7.441428 | 36.100542 | Ethiopia |
| African horse sickness | 6.4231 | 3.5689 | Nigeria |
| African horse sickness | -33.83 | 19.76 | South Africa |
| African horse sickness | -33.77 | 19.58 | South Africa |
| African horse sickness | -33.77 | 19.68 | South Africa |
| African horse sickness | -27.221 | 31.735 | Swaziland |
| African horse sickness | 7.280886 | 35.610056 | Ethiopia |
